# *Operando* Reflectance Microscopy on Polycrystalline Surfaces in Thermal Catalysis, Electrocatalysis, and Corrosion

**DOI:** 10.1021/acsami.1c04961

**Published:** 2021-04-19

**Authors:** Sebastian Pfaff, Alfred Larsson, Dmytro Orlov, Gary S. Harlow, Giuseppe Abbondanza, Weronica Linpé, Lisa Rämisch, Sabrina M. Gericke, Johan Zetterberg, Edvin Lundgren

**Affiliations:** † Combustion Physics, Lund University, Sölvegatan 14, S-22363 Lund, Sweden; ‡ Division of Synchrotron Radiation Research, Lund University, Sölvegatan 14, S-22363 Lund, Sweden; § Materials Engineering, Lund University, Ole Römers väg 1, S-22363 Lund, Sweden; ∥ Department of Chemistry, University of Copenhagen, Universitetsparken 5, DK-2100 Copenhagen, Denmark

**Keywords:** microscopy, reactor development, *operando* catalysis, *operando* electrochemistry, corrosion, mass spectrometry, cyclic voltametry

## Abstract

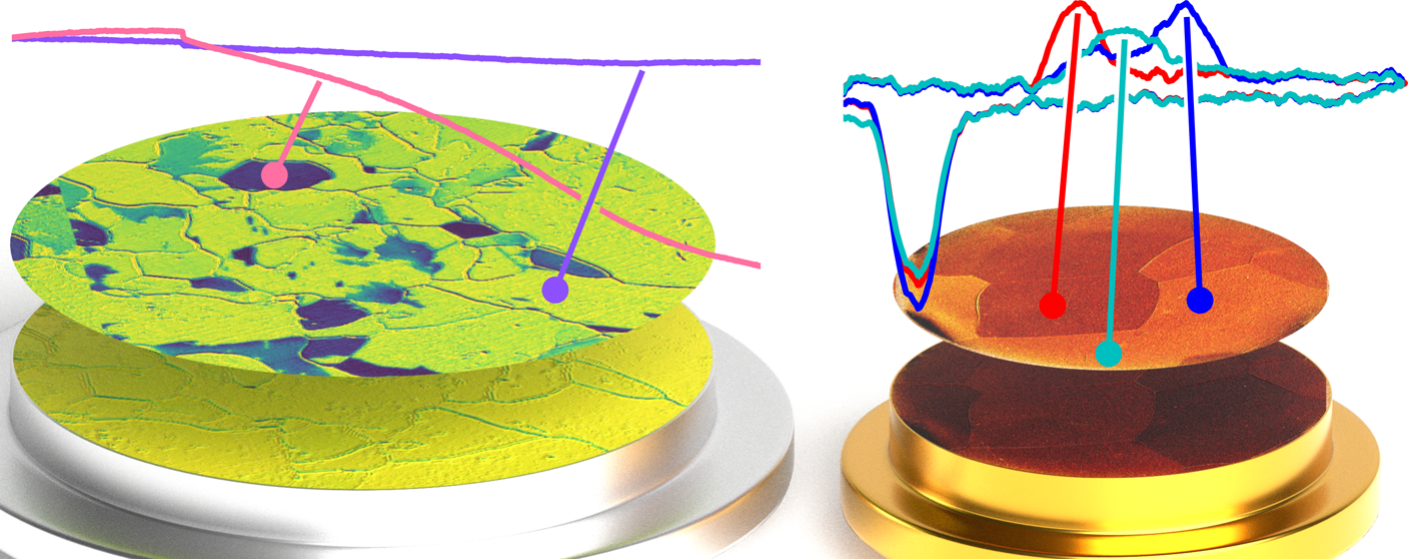

We have developed a microscope with a spatial resolution of 5 μm, which can be used to image the two-dimensional surface optical reflectance (2D-SOR) of polycrystalline samples in *operando* conditions. Within the field of surface science, *operando* tools that give information about the surface structure or chemistry of a sample under realistic experimental conditions have proven to be very valuable to understand the intrinsic reaction mechanisms in thermal catalysis, electrocatalysis, and corrosion science. To study heterogeneous surfaces *in situ*, the experimental technique must both have spatial resolution and be able to probe through gas or electrolyte. Traditional electron-based surface science techniques are difficult to use under high gas pressure conditions or in an electrolyte due to the short mean free path of electrons. Since it uses visible light, SOR can easily be used under high gas pressure conditions and in the presence of an electrolyte. In this work, we use SOR in combination with a light microscope to gain information about the surface under realistic experimental conditions. We demonstrate this by studying the different grains of three polycrystalline samples: Pd during CO oxidation, Au in electrocatalysis, and duplex stainless steel in corrosion. Optical light-based techniques such as SOR could prove to be a good alternative or addition to more complicated techniques in improving our understanding of complex polycrystalline surfaces with *operando* measurements.

## 1 Introduction

In the quest to understand complex industrial surface phenomena, there has been an increasing effort to perform *operando* studies of chemically interesting surfaces under realistic operating conditions. For heterogeneous catalysis, this means the focus moves away from ultrahigh vacuum (UHV) toward more realistic high-pressure systems;^1^ in electrochemical studies, in the fields of electrocatalysis or corrosion, it instead implies that the measurements are done while the sample is submerged in an electrolyte and a voltage is applied to the system.^2,3^ It is important to correlate changes in catalytic activity or the onset of electrochemical reaction to changes in the atomic structure and chemistry of the surface during *operando* studies. Thus, it is essential to combine surface-sensitive chemical or structural techniques with, for example, mass spectrometry (MS) in thermal catalysis or cyclic voltammetry (CV), galvanostatic, and potentiostatic techniques in electrochemistry.

Both within gas catalysis and electrocatalysis, there has been a shift toward *operando* studies of low-index surfaces, as these can serve as models for the individual facets of nanoparticle-based catalysts used in industries.^4,5^ Thus, it is now common to look at larger single crystals with (100), (110), or (111) surface orientations, as well as some stepped surfaces, where the observed chemical activity can be attributed to structures or sites on that specific surface. However, this neglects the many other possible high-index surfaces with their different kinks and steps. Also, comparing large single crystals with a huge number of orientations is both daunting and expensive.

A proposed solution to these problems is the use of samples that exhibit many surface properties to be studied simultaneously. For catalytic studies, these could be polycrystalline samples, where the surface exhibits grains of many orientations. Two-dimensional surface optical reflectance (2D-SOR) and other 2D techniques with sufficient resolution let us probe surfaces with many possible surface orientations, while monitoring the response of the individual grains.^6^

In corrosion surface science, single-phase simple alloys or single crystals have been used as model systems to gain a fundamental understanding of the corrosion process and how it relates to surface structure and chemistry.^7,8^ However, most industrial materials, such as duplex stainless steels, contain multiple phases with different physical and chemical properties. Industrial materials are almost always polycrystalline and exhibit many different surface orientations, which may behave differently under corrosive conditions. Studies of simple single-phase model systems also neglect any interactions and synergistic effects between the multiple phases, as well as the effect of grain boundaries.

Thus, within the fields of thermal catalysis, electrocatalysis, and corrosion, there is a need to study heterogeneous surfaces such as polycrystalline or multiphase materials *in situ*. This requires techniques that can provide 2D spatial information on the surface state and activity with a spatial resolution high enough to differentiate the different grains, as all grains need to be monitored separately and simultaneously. The technique must also be able to probe the sample surface through the surrounding gas and electrolyte. Techniques such as surface X-ray diffraction (SXRD) can be used both in gas phase^9^ and in electrochemistry^10^ but provide no spatial information about the surface. Others, such as low-energy electron microscopy (LEEM) and photoemission electron microscopy (PEEM), can provide 2D spatial information about the chemical state of the sample surface but are limited to UHV or high vacuum (HV) due to the low mean free path of electrons.^11−13^

Two-dimensional surface optical reflectance (2D-SOR) was introduced by Onderwaater et al.^14^ They have shown that a simple setup measuring the optical reflectance from a metal sample can be used to gain information about the sample oxide or roughness. They further stipulate that even very thin oxides with a thickness of only a few nanometers can be detected using this method.^15^ Since it works with visible light, 2D-SOR can be used under high-pressure conditions and when the sample is submerged in an electrolyte. We have since developed this method further and have also used it in combination with high-energy surface X-ray diffraction (HESXRD) where we correlate changes in the surface reflectance to changes in the surface oxide thickness and roughness on a single-crystal Pd(100) sample.^16,17^ We have shown that 2D-SOR signal is sensitive enough to detect the formation of a 2–3 Å thick surface oxide.^18^ We have also used SOR in electrochemistry to study the anodization of Al and cyclic voltammetry on single-crystal surfaces of Au(111) and Pt(100).^19^ Especially when combined with other *operando* techniques such as mass spectroscopy (MS) in gas phase or cyclic voltammetry (CV) in electrochemistry, 2D-SOR can aid in correlating changes in surface structure with changes in the chemical activity. Optical reflectance techniques have been used in the past to study local electrochemical phenomena at surfaces, such as oxidation of nanoparticles, and corrosion.^20−22^

In this work, we have further developed the possibilities of 2D-SOR by combining it with microscopy to study polycrystalline surfaces where we can resolve reflectance changes from individual grains. We believe this is a convenient and cost-effective setup to study the surface of polycrystalline metals in three important fields of surface science: gas-phase catalysis, electrocatalysis, and corrosion. To demonstrate the technique, we study CO oxidation on a polycrystalline Pd sample, cyclic voltammetry on a Au polycrystal, and cyclic voltammetry on super duplex stainless steel. Using these three examples, we show how the combination of 2D-SOR with additional techniques, can be used to follow many grains on the micron level exhibiting different surface structures simultaneously under realistic experimental conditions.

We believe that this technique makes a valuable addition to the arsenal of tools available for *operando* surface science, especially due to its ability to quickly investigate many surface orientations or surface structures at once, given its high spatial and temporal resolution. Two-dimensional SOR microscopy could be a valuable complementary technique when performing grazing incident diffraction or absorption experiments at synchrotrons.

## 2 Experimental Setup

**Figure 1:**
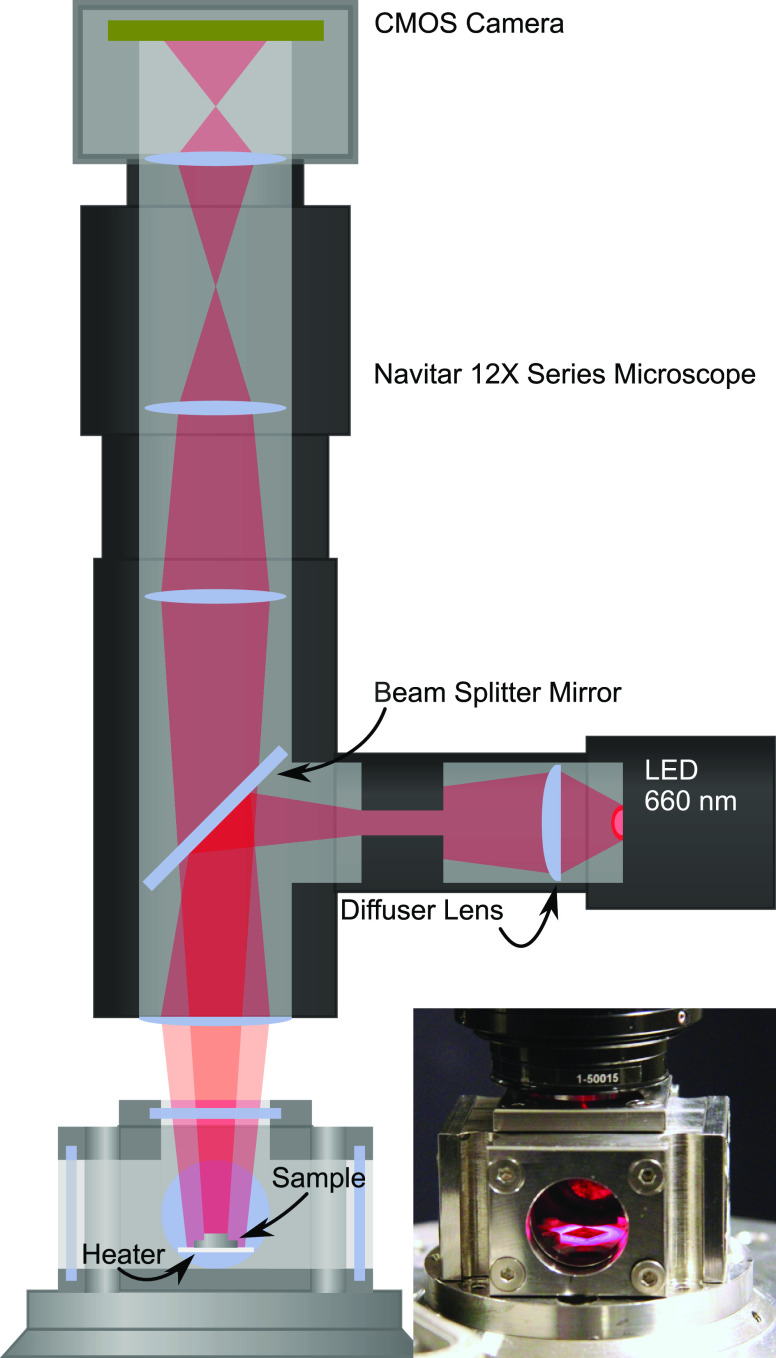
Schematic of the microscope setup and the catalytic reactor. The light source is a 660 nm LED, which is attached to a Navitar 12× microscope through the illumination port. A beam splitter directs the light to the sample surface. The reflected light is then imaged by a CMOS camera (Andor Zyla or uEye UI-3260CP). The photograph inset shows the reactor with a 6 mm × 6 mm sample placed onto the heater. The window openings are 18 mm in diameter. Note that positions of the lenses within the microscope are not accurate.

The microscope setup (Figure 1) was assembled from off-the-shelf parts and consisted of a preassembled microscopic lens system (Navitar 12× Zoom Series) with an optical illumination port. This port allowed for the attachment of a light source, the light of which was reflected onto the sample with a beam splitter. A high-intensity red light-emitting diode (LED) at 660 nm (Thorlabs M660L4) was chosen as a light source, and a diffuser lens placed between the LED and the beam splitter removed any patterns from the LED itself. The light was then reflected off the sample, and imaged using a simple 1936 × 1216 pixels, 12 bit monochrome complementary metal oxide semiconductor (CMOS) camera (uEye UI-3260CP). For the Pd measurements, we have also used a 16-bit Andor Zyla camera with similar resolution. This provides a flexible, portable, and inexpensive system, which can easily be mounted on both a reactor for heterogeneous gas-based catalysis,^17,18^ as well as on electrochemistry cells.^19^ In this work, we will treat both cases.

As a proof-of-concept, several samples which are of interest to thermal catalysis, electrocatalysis, and corrosion were investigated using the technique—a polycrystalline Pd sample used for CO oxidation, a polycrystalline Au sample for electrocatalysis and a super duplex stainless steel sample for corrosion study. These samples have surface features with sizes ranging from tens or hundreds of micrometers to several millimeters.

The Pd sample was a 2 mm thick hat-shaped polished (*r*_a_ < 0.03 μm) Pd polycrystal of 99.994% purity, with a 6 mm hat diameter and an 8 mm base diameter purchased from Surface Preparation Laboratory in Zaandam. Before the presented measurements, the sample surface was treated through three cycles of Ar^+^ sputtering and annealing to 1000 K and transferred in air to the thermal catalysis reactor. This sample was used in the gas catalysis reactor. To study the Pd sample during catalytic activity, the sample was heated under gas conditions of 40% O_2_, 4% CO, and 56% Ar, at a pressure of 150 mbar and a total flow of 100 mL_n_/min. Prior to the experiment, the sample was heated to 240 °C, which is below the critical activation temperature of Pd. The experiment itself consisted of heating the sample beyond its activation temperature at around 260 °C, and observing the resulting mass spectroscopy and SOR signals. The crystallographic orientations of the surface grains were characterized by electron backscatter diffraction (EBSD) using a scanning electron microscope (FEI Quanta 200 MKII) with an integrated camera (Hikari XP) and a TSL-OIM system from energy-dispersive X-ray analysis (EDAX).

The Pd experiment was conducted in a 23 mL high-pressure flow reactor. Optical access to the sample was provided by 18 mm diameter windows on all sides. Sample heating is done with a Boralectric resistive heater, onto which the sample is placed. The temperature of the sample was monitored with a type D thermocouple, connected to the heater. We have performed calibration measurements to map the temperature reported by the thermocouple to the sample temperature.^23^ The gas supply into the reactor is regulated with a series of mass flow controllers (Bronkhorst EL-FLOW), and a pressure controller (Bronkhorst EL-PRESS) is used to keep a constant pressure in the reactor. Using this system, we can reach flows between 10 and 500 mL_n_/min at pressures between 10 mbar and 1 bar. Pressure gauges monitor the pressure before and after the reactor, which makes it possible to determine the reactor pressure through a calibration curve. A quadrupole mass spectrometer (Pfeiffer QMP 220) into which a small amount of the exhaust gas was leaked through a leak valve was used to monitor the gas composition at the reactor outlet. More details concerning the reactor, gas system, and its capabilities can be found in refs (17, 23), and (24).

The Au sample was a 4 mm tall hat-shaped polycrystalline sample, with a hat diameter of 7.5 mm and a base diameter of 14 mm. The sample was bought polished to a mirror finish from Surface Preparation Laboratory in Zaandam. Before the electrochemical measurements, the sample was cleaned in heated concentrated nitric acid, rinsed in ultrapure water, and flame-annealed using a butane torch. On the gold polycrystal, cyclic voltammetry (CV) was performed in 0.1 M H_2_SO_4_ with a sweep rate of 20 mV/s, a lower vertex potential of −0.1 V, and an upper vertex potential of 2 V.

The super duplex stainless steel sample from Sandvik (SAF SDSS 2507) contained two phases: austenite grains in a matrix of ferrite grains. The sample was hat-shaped with a diameter of 6 mm and a base diameter of 13 mm. The sample was polished down to 0.25 μm diamond suspension followed by oxide polishing suspension (OPS) polishing. Before the experiments, the sample was cleaned in acetone, ethanol, and ultrapure water. To study the behavior of the different phases of the steel in a corrosive environment, CV in 1 M NaCl was performed with a sweep rate of 50 mV/s, an upper vortex potential of 1.5 V, and a lower vortex potential of −0.9 V. To resolve the grains of the super duplex stainless steel sample a 1.5× magnification lens was used in combination with the Navitar microscopic lens system.

**Figure 2:**
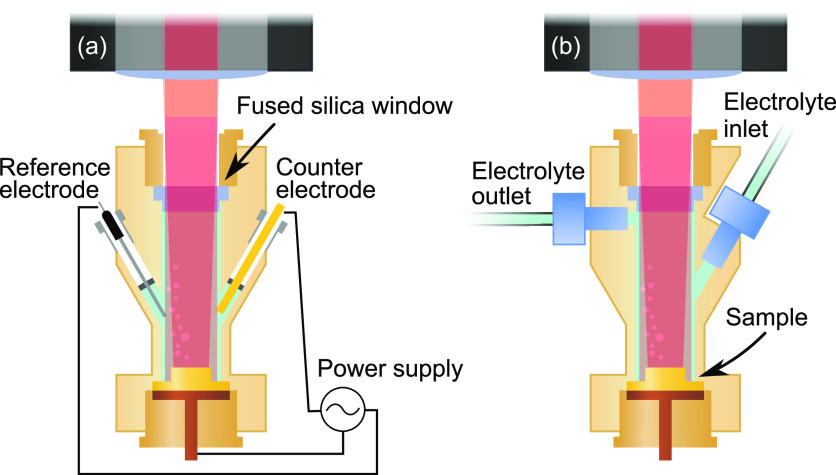
Electrochemistry cell. (a) Cross section of the cell where the electrodes are visible. (b) Cell rotated 90°, where the electrolyte inlet and outlet are visible. Note how the fused silica window protrudes into the cell, ending at the same height as the electrolyte outlet. Thus, any bubbles that would distort the SOR image can be pumped out from the cell.

The electrochemistry cell used for the Au and super duplex stainless steel experiments was a polyether ether ketone (PEEK) flow cell with a three-electrode system as shown in Figure 2. The counter electrode was a gold rod and the reference electrode was a saturated Ag/AgCl electrode (eDAQ, ET072). A peristaltic pump was used to circulate the electrolyte from a bottle through the cell. The sample and optical window were sealed using Kalrez O-rings. The cell is described in more detail elsewhere.^19,25^ A custom-made fused silica window provided optical access to the sample surface. The silica window was made in a top hat shape to let the glass protrude into the cell leaving the bottom of the glass window at the same height as the electrolyte outlet, as can be seen in Figure 2b). This is an improvement on the previous design,^19^ as there is now no volume above the electrolyte outlet to trap gas in the form of bubbles. These bubbles, which disturb the 2D-SOR image, often originate from electrochemical reactions on the surface such as the oxygen evolution reaction (OER) or the hydrogen evolution reaction (HER). The lower positioning of the glass window also allowed for the optics of the SOR microscope to be placed closer to the sample, enabling higher optical magnification.

## 3 Results

**Figure 3:**
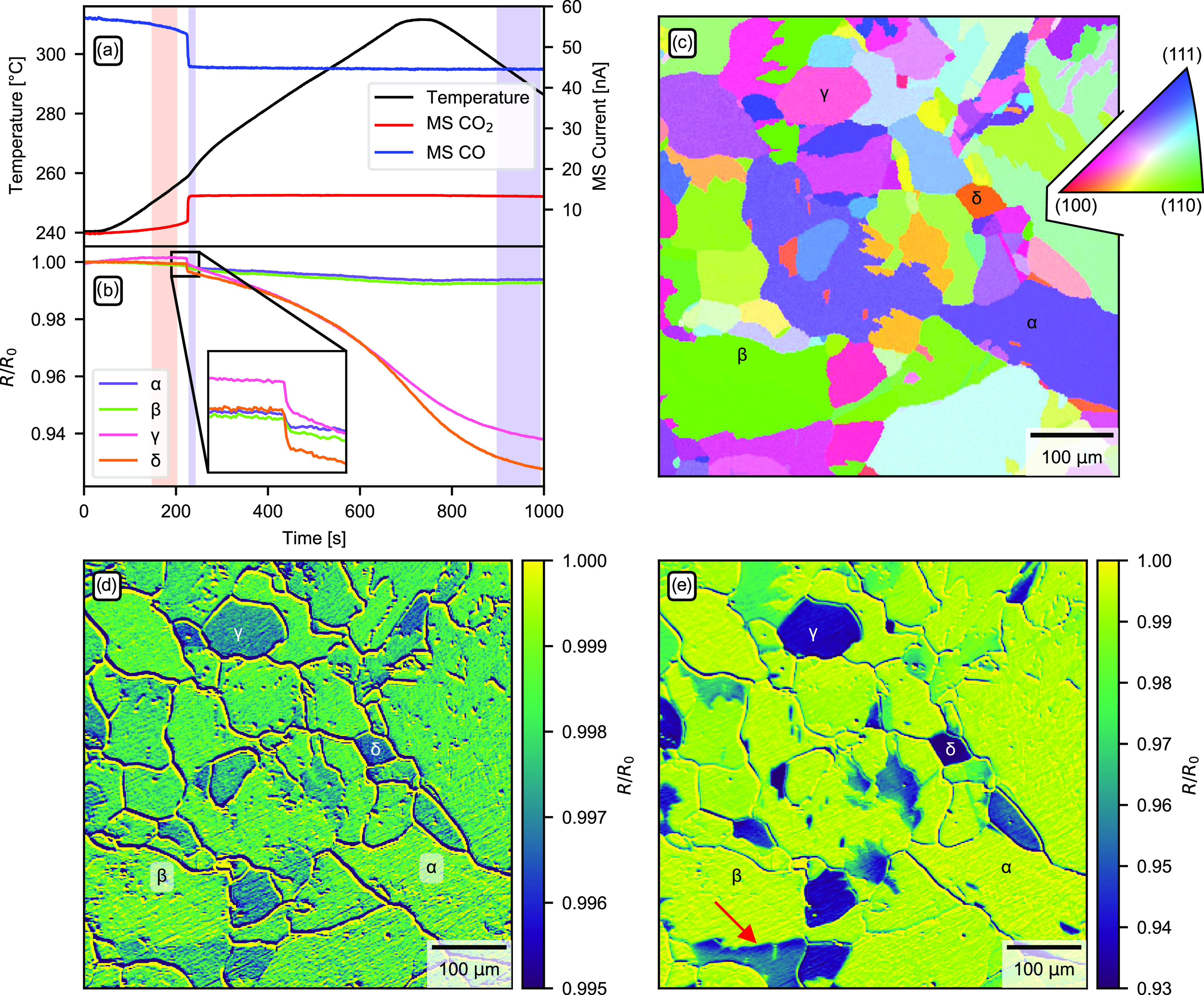
Catalytic CO oxidation over a polycrystalline Pd catalyst. (a) Mass spectrometer and temperature trends as the Pd sample is heated above its activation temperature. (b) Relative reflectivity change as measured with SOR for a number of grains marked with the letters α, β, γ, and δ in (c)–(e). (c) EBSD map of the sample, revealing the local grain surface orientations. (d, e) Reflectivity change of the sample surface at the left and right blue regions marked in (a), respectively. Both (d) and (e) show the change in reflectivity with respect to the red region in (a). Note that grains α and β remain highly reflective throughout the measurement, while grains γ and δ become much less reflective. The red arrow indicates a spot where a grain exhibits a reflection intensity gradient. A video is available in the Supporting Information.

The results from the experiments involving the polycrystalline Pd sample in the gas catalysis reactor are shown in Figure 3. During the experiment, the sample was heated from 240 to 300 °C in 700 s as shown in panel (a). At a sample temperature of around 260 °C, the sample enters a high-activity region where the catalytic activity is limited by the diffusion of gas to the sample instead of the intrinsic activity of the sample surface, also known as mass transfer limit (MTL). This is evident from the mass spectrometer trends leveling off, even though the temperature is increased further. Simultaneously to monitoring the exhaust gases with a mass spectrometer, the reflectivity of the surface grains was monitored using SOR.

A number of grains (denoted α, β, γ, and δ) were selected and their relative reflectance change is shown in Figure 3b. Panel (c) shows an EBSD measurement of the area of interest, with the aforementioned grains labeled. This shows that grain α is quite close to a (112) orientation at (11 19 12), grain β is quite close to a (110) orientation at (20 17 1), and grains γ and δ are close to (100) at (9 5 32) and (4 22 1), respectively. The SOR data are shown in panels (d) and (e). Panel (d) shows the reflectivity of the sample just after the light-off where the sample reaches the high-activity regime as denoted by the left blue region in panel (a). Panel (e) shows the reflectivity after the sample has been active for around 10 min, as denoted by the right blue region in panel (a). Note that the color scales are very different on the two images; while the reflectivity of the grains decreases at most around 0.5% just after the light-off as shown in panel (d), the reflectivity has decreased 7% after the sample has been active for a few minutes as shown in panel (e). In both panels (d) and (e), the images have been normalized using the inactive sample as a reference, as denoted by the red region in panel (a).

From Figure 3d,e, it is evident that grains with different crystallographic surface orientations behave very differently. While grains closer to (100) seem to become considerably less reflective, grains closer to (110) or (111) change less. It should also be noted that there is an initial “jump” in reflectivity loss as the grains transition into the active regime, as shown in the magnified region of panel (b). This is consistent with previous studies^17,18^ and may be due to a thinner layer of oxide forming as the gas composition changes when the reactant gases are depleted by the chemical reaction. Other details of note are the spatial gradients visible on the edges of some of the grains in panel (e), for example, the one at the tip of the red arrow. These could be attributed to gas diffusion as different grains may have different catalytic activity and thus the local gas composition may differ between grains creating a diffusion gradient at grain boundaries. The effect may also be attributed to enhanced reactivity at the grain boundaries themselves, since atoms across several layers in their vicinity are displaced from their typical low-energy lattice sites.

**Figure 4:**
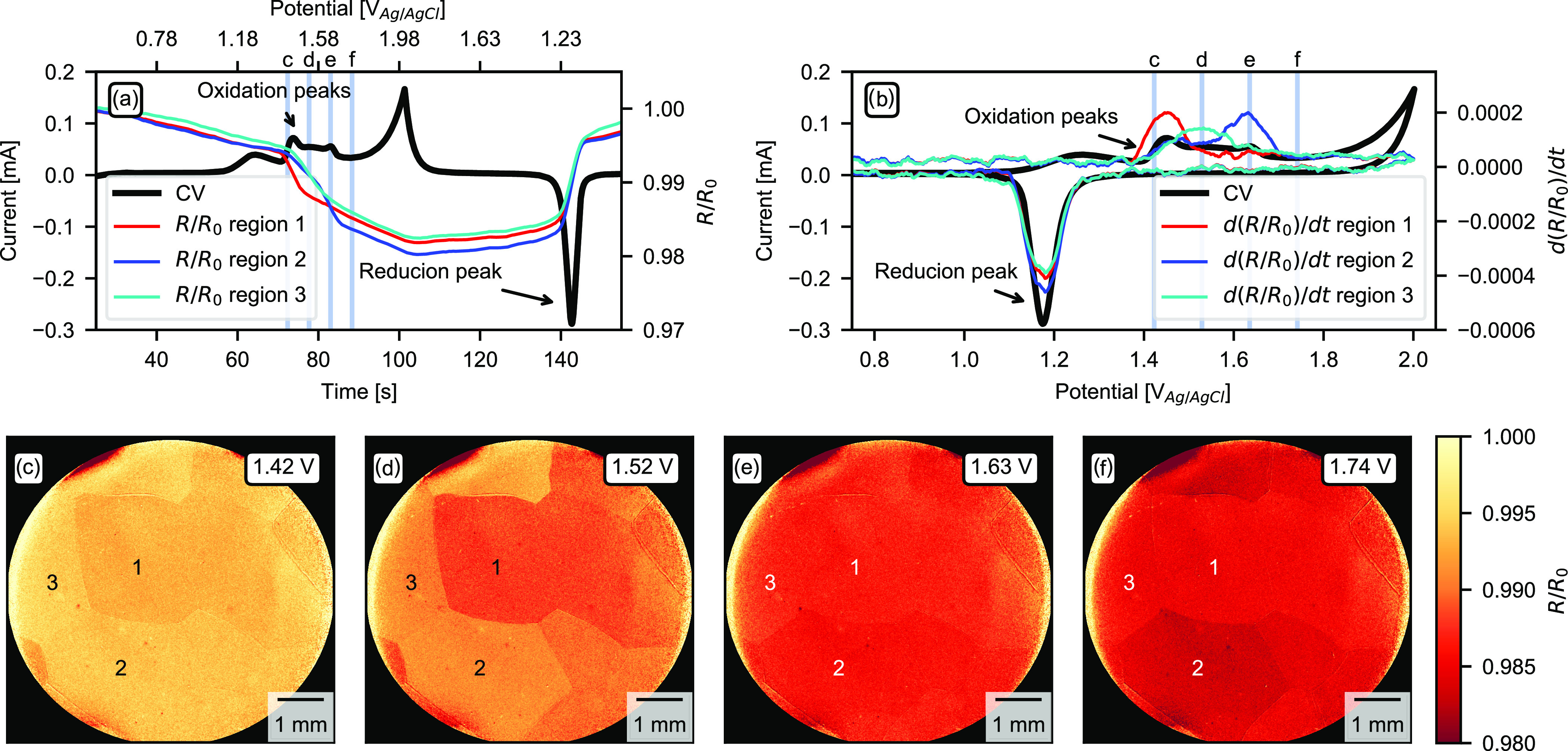
CV on Au polycrystal in 0.1 M H_2_SO_4_. (a) CV current as a function of time, overlayed with reflected intensity from grains 1–3 shown in (c)–(f). (b) CV current as a function of potential, overlayed with the derivative of the reflected intensity from grains 1–3 shown in (c)–(f). (c–f) Two-dimensional normalized SOR images showing the reflected intensity of the grains during the oxidation peaks in the CV. Position of potentials marked with blue lines in (a) and (b). A video is available in the Supporting Information.

Results from CV on the Au polycrystal in 0.1 M H_2_SO_4_ are summarized in Figure 4. The SOR data were normalized on a per pixel basis to the intensity at time zero. Panel (a) shows the normalized reflected intensity from the surface of different grains in the sample, indicated by 1–3 in Figure 4c. Note that the grains are several millimeters in size. The normalized reflectance is also plotted together with the current from the CV as a function of time. The positive current peaks (anodic peaks) that can be seen in the CV correspond to oxidation reactions at different gold surfaces.^26,27^ There is a rapid decrease in the reflected intensity when the oxidation peaks are reached in the CV. A clear increase in the reflected intensity is seen, coinciding with the position of the reduction peak in the CV.

The current in the CV is a flow of electrons that is proportional to the change rate of the system and not the absolute state of the system. In contrast, the reflected light intensity is proportional to the roughness and optical properties of the surface. Thus, the SOR intensity is proportional to the absolute state of the system and not the change rate of the system. This means that the time derivative of the SOR signal corresponds to the change in the state of the system and can be compared to the current in the CV. Panel (b) shows the CV plotted together with the derivative of the SOR signal from three grains on the polycrystalline gold surface. Taking the derivative of the SOR signal results in peaks around a constant background, closely matching the current of the CV. It can also be seen that the peaks of the SOR derivative overlap with the oxidation and reduction peaks of the CV. However, there is no drastic change in the SOR signal during the oxygen evolution reaction (OER) at around 2 V. There is also no peak in the SOR derivative matching the peak of the OER at high potentials. This indicates that within the potential window of the experiment, there is no significant additional roughening or oxidation of the sample surface during the OER.

Figure 4c–f shows the normalized 2D SOR images at different potentials around the oxidation peaks. The potentials are marked with blue lines in panels (a) and (b). From the SOR images, it is clear that the different grains behave differently during the electrooxidation. The grains exhibit different kinetics and require different overpotentials for the reactions to take place. The current measured during the CV is the superposition of the contribution of all of the different grains on the surface of the crystal. With the help of the spatially resolved SOR microscope, we can now deduce the contribution to the CV from the different grains. It is well known in the literature that the three different close-packed surfaces (100), (110), and (111) of gold have different cyclic voltammograms in a H_2_SO_4_ electrolyte.^27^

**Figure 5:**
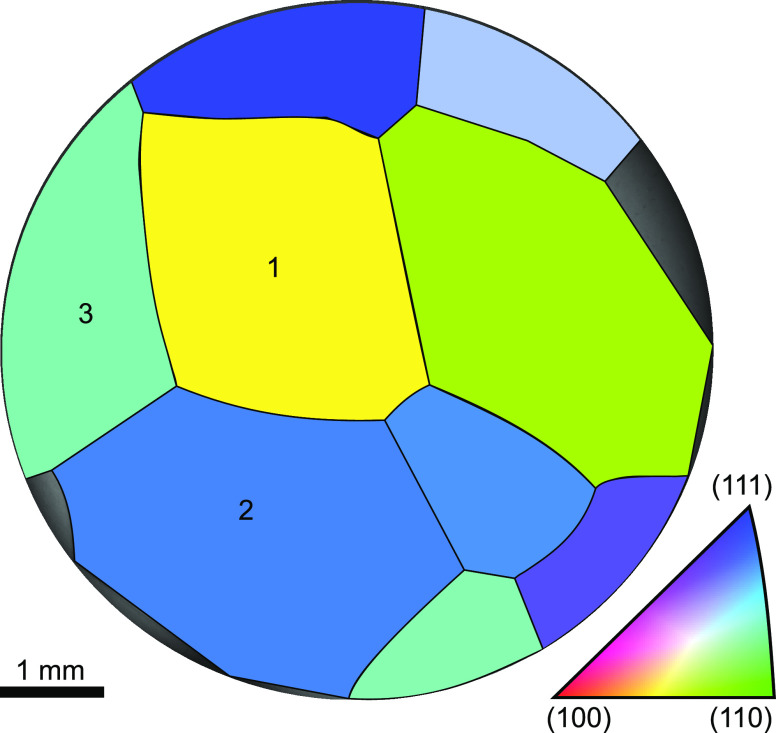
EBSD image of the Au polycrystal. The numbering here matches that in Figure 4. We can see that region 1 is right in between (100) and (110), which is a (210) surface. Grain 2 is rather close to (111), whereas grain 3 is between (110) and (111), resulting in a (221) surface.

From the EBSD data shown in Figure 5, the surface orientation of the grains can be determined. The surface of grain 1 is oriented between the (100) and (110) direction, which correspond to a (210) surface, which exhibits small (100) terraces and (110) steps. The reflectance voltammogram recorded with SOR from grain 1 is similar to the CV of a (110) Au single-crystal surface.^27^ An interpretation of this is that the (110) steps are primarily oxidized at the (210) surface. The orientation of grain 2 is very close to (111) and the reflectance voltammogram of grain 2 is very similar to the CV of Au(111) electrodes from the literature.^27^ Grain 3 has a crystallographic orientation between the (111) and (110) direction, which correspond to a (221) surface in the stereographic triangle. This surface has (111) terraces and (111) steps. According to the SOR signal shown in 4, the onset of oxidation for grain 3 occur at a lower potential than for grain 2. Both have the (111) character, but the high-index surface of grain 3 has a higher step density. This indicates that the step atoms are less stable during electrooxidation, and oxidize at lower potentials than the (111) terrace atoms. In the gas phase, it is known that steps have a lower energy barrier for oxidation than the atoms at the flat terraces.^28,29^ The surface stability determined from the combination of 2D-SOR, CV, and EBSD follows the trend where the most uncoordinated surface atoms are oxidized at the lowest potentials. The coordination number of surface atoms for an face-centered cubic (FCC) crystal goes in the order of 7 for (110), 8 for (100), and 9 for (111). At the (210) surface of grain 1, the (110) step atoms are the least coordinated and seem to oxidize first. The (221) surface contains (111) terraces and (111) steps, where the step atoms are less coordinated than the terrace atoms, and hence oxidize at lower potentials than the (111) terrace atoms. The order of oxidation onset of the grains: 1, 3, and 2 can be attributed and correlated to the coordination number of the least stable surface atoms. Studies performed on electrooxidation of platinum, which is a similar system, showed that oxidation roughens the surface and lift surface atoms from their equilibrium positions.^2,30^ This would result in a decrease in reflected intensity just as observed in this study. For platinum, it was also observed with HESXRD that less coordinated surfaces oxidize at lower potentials.^2^

With the SOR microscope, we can study different grains at the surface exposed to the same experimental conditions. This makes the comparison of different surface orientations and surface structures more reliable since the external experimental conditions such as electrolyte concentration and composition as well as temperature and potential can be kept the same for all grains on the polycrystalline surface.

In electrochemistry, polycrystalline surfaces are often used as working electrodes. Here, we can clearly see that the current observed with electrochemical techniques is composed of the sum of the contribution of each separate grain. This means that the crystallographic orientations present at the surface determine the electrochemical properties since they in turn depend on the atomic structure of the surface. A polycrystalline surface may hence have very different electrochemical properties if there is any preferential orientation or texture of the grains at the surface.

**Figure 6:**
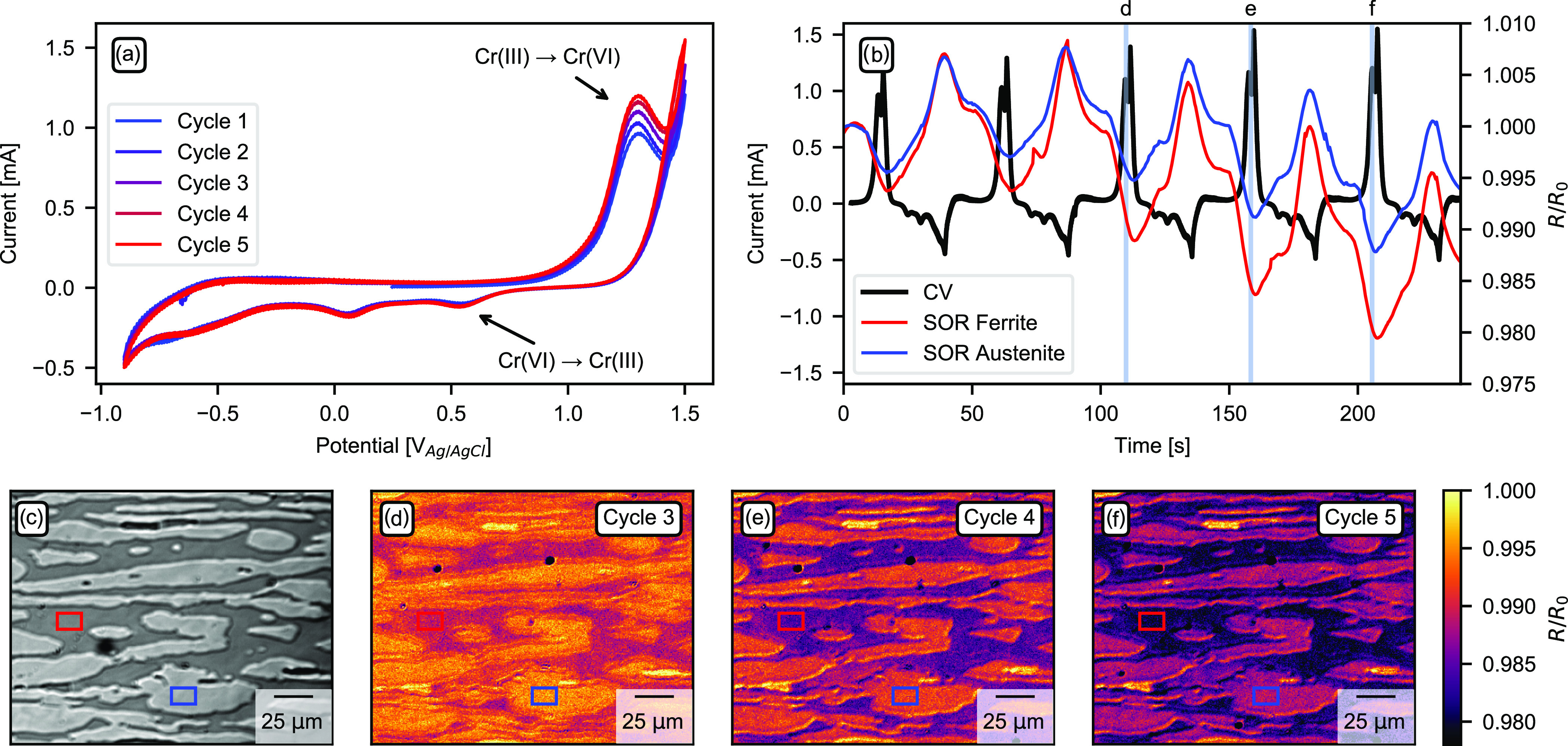
Measurements from the super duplex stainless steel sample. (a) CV on super duplex stainless steel in 1 M NaCl. (b) CV current as a function of time overlayed with reflected intensity of ferrite and austenite from the areas marked with a red and blue box, respectively, in (c)–(f). (c) Two-dimensional SOR image prior to the corrosion experiment. Bright areas correspond to the austenite phase, and darker areas correspond to the ferrite phase. (d–f) Two-dimensional normalized SOR images showing the reflected intensity of the two phases during Cr(III) oxidation. Positions of the images are marked with blue lines in (b). A video is available in the Supporting Information.

Results from the super duplex stainless steel corrosion experiment are summarized in Figure 6. The 2D-SOR data were normalized on a per pixel basis to the intensity at time zero. Panel (a) shows the five cycles of the CV in 1 M NaCl. As can be seen, the anodic peak at around 1.4 V, which is attributed to the oxidation of Cr(III) to Cr(VI),^31−33^ grows in current density with each cycle. The current density of the cathodic peak at around 0.5 V, which corresponds to the reduction of Cr(VI) to Cr(III),^31−33^ does not increase as the cycles proceed. With every cycle, more Cr gets oxidized during the anodic sweep but is not reversibly reduced during the cathodic sweep. This indicates that the dissolution rate of Cr from the passive film increases with each sweep.

Figure 6b shows the current from the CV as a function of time plotted together with the normalized SOR intensity from the surface of the different phases, austenite and ferrite. As seen, during the anodic peak in the CV corresponding to the oxidation of Cr(III), the intensity of the SOR signal decreases. Upon reduction of the formed Cr(VI), the intensity of the SOR signal increase. However, when the SOR intensity increases upon the reduction of Cr(III), the SOR intensity does not return to the corresponding intensity of the previous cycle. There is a decay in the overall SOR signal over time as the sample is cycled. Also note that the ferrite phase SOR intensity decreases more than that of the austenite phase. The SOR signal from the two different phases is very similar except that the signal from the ferrite phase deteriorates faster than that of the austenite phase.

Figure 6c shows a raw SOR image of the super duplex stainless steel surface before the corrosion experiment. The bright areas are the austenite phase, the darker areas are the ferrite phase. Panels (d)–(f) show the normalized SOR images after cycles 3–5 extracted from the regions marked with a blue line in panel (b). From the images, it can be seen that the ferrite matrix deteriorates faster than the austenite grains within the whole measurement area. It is known from the literature that for duplex stainless steel, the ferrite matrix corrodes at a higher rate than the austenite.^34^ Due to the differences in crystal structure and elemental composition between the two phases, the passive film on the surface also differs between austenite and ferrite.^35^ A change in the reflected intensity is caused either by a change in surface roughness or by a change in the optical properties of the surface. Here, we can observe a decrease in the SOR signal that corresponds to the oxidation of Cr(III) to Cr(VI), which seems to be the mechanism of degradation of the passive film and later the metal itself. According to our SOR measurements, Cr(III) oxidation and dissolution is more prominent at the ferrite phase, which might be caused by a different oxide chemistry or structure. With our 2D SOR microscope, it is possible to monitor the corrosion of real industrial materials *in situ*, submerged in the electrolyte, and under potential control. Measurements can be performed in real time to catch the dynamics and behavior of individual grains down to tens of micrometers in size.

## 4 Discussion

We have presented three examples where 2D-SOR was used to gain valuable insights into surface phenomena, demonstrating the strengths of 2D-SOR as a surface science tool. We have shown that 2D-SOR can be used to image both small areas of a polycrystalline sample, with spatial resolutions of 5 μm as was the case for the Pd and super duplex stainless steel measurements, as well as entire samples as demonstrated in the Au measurement with a 7 mm field of view. We have further shown that 2D-SOR can resolve very small changes in reflectance of less than 0.1%, as demonstrated in Figure 3d. We used exposure times down to 10^–5^ s without sacrificing signal-to-noise ratio. Thus, the temporal resolution is primarily limited by the readout time of the CMOS sensor, and studies with much higher temporal resolution than demonstrated in this work are possible, given that a suitable camera is used. As was mentioned in the Section 2, the 2D-SOR setup, including the uEye camera, costs around 7000€. This makes the technique available to a broad range of surface science researchers. The compact design also allows it to be easily transported to large-scale facilities, such as synchrotrons, where it can be used to complement other *operando* techniques. Another advantage of 2D-SOR is that that the 2D spatial resolution enables *operando* studies of heterogeneous surfaces. This is an important step that allows for *operando* studies to move away from single-crystal model systems. Well-controlled single-crystal studies have been vital for a fundamental understanding of surface science, both in the fields of catalysis and corrosion. Thus, with 2D-SOR, heterogeneous surfaces more closely resembling those used in industrial applications can be studied.

In the field of surface science, there are few tools compatible with high gas pressures or a presence of an electrolyte that provide 2D spatially resolved data. Techniques that use X-rays, such as HESXRD or surface-sensitive X-ray absorption spectroscopy (XAS), can probe surfaces in *operando* conditions, but provide no spatial information about the surface. Electron-based techniques, such as PEEM, provide spatially resolved information but are difficult to operate at high gas pressures or in an electrolyte, necessary for *operando* measurements. Scanning probe techniques can be performed *in situ*, such as electrochemical scanning tunneling microscopy (EC-STM), which can provide spatial structural information at the atomic scale while electrochemical reactions take place at the surface.^30^ However, EC-STM is very cumbersome to perform and is limited to a small field of view. Further, electron and X-ray-based techniques can be intrusive and unwillingly alter the surface to be studied due to beam-induced damage. As demonstrated in this work, 2D-SOR provides spatially resolved information about the sample surface in a nonintrusive manner, and is compatible with both high gas pressures and the presence of an electrolyte. Another 2D-capable technique which has been employed in electrochemistry is second-harmonic generation imaging (SHGI) which has previously been used to investigate a polycrystalline Au surface during CV.^36^ This technique is sensitive to the surface charge density which can be visualized as a function of the applied potential. Comparing 2D-SOR to SHGI, we can see that both techniques provide spatially resolved surface-sensitive data. The main advantage of 2D-SOR is the capability to use the technique in both electrochemistry and thermal catalysis, whereas SHGI can only be used in situations where there is a potential between the surface and the surrounding medium. Furthermore, the 2D-SOR setup is relatively inexpensive and simple compared to SHGI. SHGI on the other hand provides information which is easier to quantify and allows for distinguishing oxide formation from roughening. Thus, 2D-SOR microscopy helps to fill a gap in the techniques available in the surface scientist’s toolbox.

In the case of the electrochemistry application, the advantage of 2D-SOR is clear. We can determine the shape of the CV on a per grain basis, and hence, we can learn what electrochemical reactions take place at each grain. To verify this, we can compare the average change in reflectivity over the whole sample to the CV, which shows which features in the CV can be detected by 2D-SOR. We can also follow to what extent each grain is affected by the electrochemical reactions as in the case of the steel, where irreversible changes occurred due to oxidation of Cr(III) which was more prominent for the ferrite phase. For the case of the gold surface, we showed that we can image the surface oxidation as a function of potential. The local CV for each grain can be correlated to the CV of the low-index gold surfaces from the literature. This information can be compared to the crystallographic orientation measured with EBSD. In summary, we could disentangle the contribution of the CV from the different grains on the surface. In the case of the super duplex stainless steel, we could observe that the changes to the surface structure corresponded to the oxidation of Cr(III) to Cr(VI) and that the ferrite phase corrodes faster than the austenite phase.

In the case of gas-phase catalysis exemplified with the Pd measurement, the learning outcome is more subtle. The primary limitation of 2D-SOR is that the technique is not quantitative on its own, which makes it difficult to distinguish if a change in reflectivity is caused by roughening, oxidation or both. Instead, we can use 2D-SOR to observe which grains exhibit a change in any of these properties, and which do not. We can further use 2D-SOR to learn about the magnitude and speed of this change. The real strength of 2D-SOR, however, becomes apparent when combining 2D-SOR with other *operando* capable techniques. By combining the 2D-SOR with MS in this work, we show how the surface orientation of the individual grains affects the oxidation behavior during catalytic CO oxidation, and correlate changes in the grains to changes in overall catalytic activity. Furthermore, there are many other techniques that can determine the exact surface oxide properties. For example, high-energy X-ray diffraction (HESXRD) can determine the oxide structure. X-ray reflectivity (XRR) can be used in conjunction with that to determine the oxide thickness. Measurements with 2D-SOR combined with HESXRD on single crystals have been performed, and it was observed that slow changes in the reflectivity correlate primarily with oxide formation, whereas slow changes correlate primarily with roughening.^18^ In this work, it was also shown that 2D-SOR is sensitive even to very thin surface oxides and that a 30 Å oxide layer results in a 10% reduction of the 2D-SOR intensity. The issue with the techniques mentioned above is that they do not provide spatial resolution which is required to distinguish individual grains. Therefore, the combination of 2D-SOR and any grazing incidence X-ray technique is a powerful way to study surfaces of complex materials systems under working conditions.

To obtain spatially resolved chemical and structural information, the 2D-SOR setup could be extended to use several LEDs of different wavelengths, which would illuminate the sample consecutively. This would allow the acquisition of spectrally resolved images, albeit with a spectral resolution limited by the number of LEDs and their respective bandwidth. The result, however, would be a hybrid of 2D-SOR and electroreflectance, which has been used to study oxides since the 1960s.^37^ In such a configuration, any roughening should decrease the reflectivity similarly for all wavelengths, whereas the dielectric properties of the surface would change due to oxide formation and change the reflectivity differently for different wavelengths. Such a multispectral approach is a natural continuation of this work. It may also be possible to add polarizers to the setup, yielding a spatially resolved variant of ellipsometry.

On top of these possible extensions of 2D-SOR itself, we plan to perform planar laser-induced fluorescence (PLIF) measurements on samples with slightly larger grains, where we hope be able to measure the actual catalytic activity on a per grain basis. This would build on previous studies where PLIF has been combined with 2D-SOR on single crystals.^16,17^ It would then be possible to directly correlate a local change in the reflectivity with a local change in catalytic activity.

As mentioned above, 2D-SOR can be used as another simple-to-use technique together with techniques such as HESXRD to correlate 2D-SOR data to changes in the atomic structure of the surface. It can also be combined with X-ray reflectivity (XRR) to correlate the 2D-SOR signal to changes in surface roughness or oxide film thickness.^18^ Combining 2D-SOR with surface-sensitive XAS could enable the correlation of changes in reflectively to changes in the chemical state of the surface, for instance upon oxidation in gas phase or in electrochemistry. Two-dimensional SOR could also be used to study localized phenomena on surfaces. In corrosion, local corrosion events, such as pitting corrosion and stress corrosion cracking could be studied *in situ* for industrially relevant materials. Pitting corrosion is a problem both for structural materials such as steels^38,39^ and aluminum alloys but also for the stability of electrocatalysts^40^ and has previously been studied^41,42^ using optical imaging. Another application of 2D-SOR could be to study the dynamics of surface oxidation, which is of relevance in corrosion protection and catalysis. Together with complementary techniques, 2D-SOR could be used to draw conclusions about how the crystallographic orientations at the surface influence the orientation, crystal structure, and growth kinetics of surface oxides. Finally, 2D-SOR microscopy and other spatially resolved techniques could aid in the creation of surface structure libraries, due to their ability to quickly study many different surface structures en masse. One measurement could provide data on several hundred surface orientations. These libraries could, for example, be used as a training dataset for machine learning algorithms to increase our fundamental understanding of processes at surfaces in *operando* conditions.

## 5 Conclusions

Few traditional surface science techniques that work in *operando* conditions provide spatially resolved data. However, spatial resolution is essential to characterize and understand polycrystalline or heterogeneous surfaces with many different crystallographic surface orientations. In this work, we have shown that the 2D-SOR microscopy technique, with high temporal resolution and with a spatial resolution of 5 μm, can be used as an *operando* technique to monitor polycrystalline surfaces on a per grain basis during reactions in gas-phase catalysis and electrochemistry. We can correlate each grain in the SOR image to a surface orientation as determined by EBSD. While this method cannot definitely determine if the change in reflectance is due to roughness or oxidation, previous HESXRD measurements indicate that slow changes in the surface reflectance correlate primarily to roughness while rapid changes correlate primarily to oxidation.^18^ This shows that the technique can be used to draw a qualitative conclusion when combined with other techniques. We believe that 2D-SOR microscopy can serve as a great complement to traditional surface science techniques, since it is nonintrusive and can provide 2D spatial information on heterogeneous surfaces such as polycrystalline surfaces. Since the technique uses light in the visible range, SOR can penetrate gas and electrolyte, which is difficult with traditional electron-based surface science techniques. Further, data from mass studies of surfaces made possible by 2D-SOR could be used as a training dataset for machine learning algorithms. This would allow us to draw conclusions from correlating data from many surface orientations measured with SOR and complementary techniques. This knowledge in turn could be used to tailor the chemistry and structure of industrially relevant surfaces in catalysis and corrosion for the needs of tomorrow.

## Supporting Information Available

Video of the Pd experiment, which is illustrated in Figure 3 (MP4)
Video of the Au experiment, which is illustrated in Figure 4 (AVI)
Video of the steel experiment, which is illustrated in Figure 6 (AVI)

## Supplementary Material

am1c04961_si_001.avi

am1c04961_si_002.avi
